# Preparation, anti-biofouling and drag-reduction properties of a biomimetic shark skin surface

**DOI:** 10.1242/bio.016899

**Published:** 2016-03-03

**Authors:** Xia Pu, Guangji Li, Hanlu Huang

**Affiliations:** 1 School of Materials Science and Engineering, South China University of Technology, Guangzhou 510640, China; 2 College of Chemistry and Chemical Engineering, Zhongkai University of Agriculture and Engineering, Guangzhou 510225, China

**Keywords:** Shark skin, Microstructure, Anti-biofouling, Biomimetic

## Abstract

Shark skin surfaces show non-smoothness characteristics due to the presence of a riblet structure. In this study, biomimetic shark skin was prepared by using the polydimethylsiloxane (PDMS)-embedded elastomeric stamping (PEES) method. Scanning electron microscopy (SEM) was used to examine the surface microstructure and fine structure of shark skin and biomimetic shark skin. To analyse the hydrophobic mechanism of the shark skin surface microstructure, the effect of biomimetic shark skin surface microstructure on surface wettability was evaluated by recording water contact angle. Additionally, protein adhesion experiments and anti-algae adhesion performance testing experiments were used to investigate and evaluate the anti-biofouling properties of the surface microstructure of biomimetic shark skin. The recorded values of the water contact angle of differently microstructured surfaces revealed that specific microstructures have certain effects on surface wettability. The anti-biofouling properties of the biomimetic shark skin surface with microstructures were superior to a smooth surface using the same polymers as substrates. Moreover, the air layer fixed on the surface of the biomimetic shark skin was found to play a key role in their antibiont adhesion property. An experiment into drag reduction was also conducted. Based on the experimental results, the microstructured surface of the prepared biomimetic shark skin played a significant role in reducing drag. The maximum of drag reduction rate is 12.5%, which is higher than the corresponding maximum drag reduction rate of membrane material with a smooth surface.

## Abstract (precis)

**Summary:** Specific microstructures of the shark skin surface have certain effects on surface wettability, and play a key role in reducing drag and anti-biofouling properties.

## INTRODUCTION

Bionics has been defined as biologically inspired design which has been adapted or derived from nature (Bhushan, 2009). In recent years, considerable progress has been made in the design and manufacture of nanomaterials, nanodevices, functional surfaces, etc. by mimicking biology and nature (Bhushan, 2009; Dean and Bhushan, 2012; Lang et al., 2014). A fine example of bionic design is the biomimetic drag reduction that is achieved using a special microstructure surface which emulates the non-smooth surface of certain natural organisms. Such an example is the shark skin effect, which is defined as a mechanism of fluid drag reduction by a riblet structured surface similar to that of the sharks' skin (Bechert et al., 1997). Sharks' skin has been extensively studied for decades because of its drag-reduction and antifouling properties (Friedmann et al., 2010; Han and Zhang, 2008; Kesel and Liedert, 2007).

With the hope of further improving drag reduction, the various microstructures on the surface of the skins of fast-swimming sharks have been examined and analysed. The skin of shark is covered by minute individual tooth-like scales termed dermal denticles, which are ribbed with longitudinal grooves which are aligned in the water flow direction. The dermal denticles on the surface of a shark's skin form an interlocking array (Reif, 1985). In order to clarify the effects of the non-smooth surface of a shark's skin on drag reduction, biomimetic surfaces with microstructures similar to those of various sharks have been designed and manufactured, thus, different approaches for fabricating these surfaces have been explored for decades. These approaches include surface machining (Walsh, 1982), laser etching (Bixler and Bhushan, 2013), photolithography (Schumacher et al., 2007), grinding and rolling (Denkena et al., 2010; Hirt and Thome, 2008), 3D printing (Wen et al., 2014) and bio-replicated forming, etc. (Chen et al., 2014). Overall, substantial progress has been made in the fabrication of biomimetic riblet-structured surfaces based on the drag-reduction properties of shark skin. Nevertheless, the techniques developed are not yet capable of meeting the requirements of large-scale fabrication. The manufactured surfaces have been evaluated in terms of their drag reduction capability (Dean and Bhushan, 2010; Oeffner and Lauder, 2012). Compared with the flat surface which served as a control, the biomimetic surfaces reduced the fluid flow drag in fluid flow by 5% to 10% (Bechert et al., 1997, 2000; Walsh and Lindemann, 1984; Zhao et al., 2012). A non-smooth shark skin surface with special microstructures is an excellent natural template for a low-drag surface.

Up to now, most investigations on the biomimetic non-smooth surface of shark skin have focused on its riblet structures. However, understanding the drag-reduction effects and anti-biofouling properties of non-smooth surfaces has proven to be very complicated. Especially, as it can be affected by various factors, such as roughness, hydrophilicity, and hydrophobicity of non-smooth surfaces (Feng et al., 2002). In this study, scanning electron microscopy (SEM) was used to examine the surface microstructure and fine structure of shark skin and biomimetic shark skin. Additionally, the surface microelements of shark skin were analysed. Specifically, in order to analyse the hydrophobic mechanism of shark skin surface micro-structure, the Wenzel model of hydrophobic theory was used to simulate the relationship between the surface microstructure and hydrophobic property of the biomimetic shark skin. In addition, protein adhesion experiments and anti-algae adhesion performance testing experiments were performed to investigate and evaluate the antifouling property of the surface microstructure of biomimetic shark skin. Drag-reduction effect of the prepared biomimetic shark skin was also tested.

## RESULTS

### Surface morphology

**Fig. 1.**
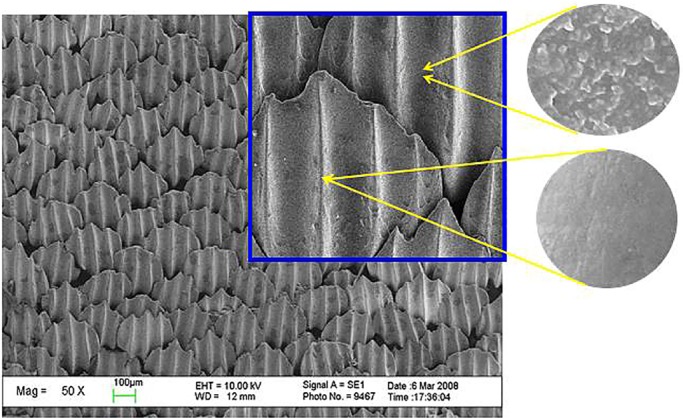
**SEM images of the riblet structures of shark skin surface.** The scale-shaped denticles form an interlocking 3D arrangement along the longitudinal axis of a shark's body. The fine structures of the denticles were also carefully observed. The surface morphologies of the raised ridge and the concave groove of the denticle were shown, respectively. The ridge possesses relatively smooth surface structure. However, some nanostructured protuberances were found on the concave groove surface.

**Fig. 2.**
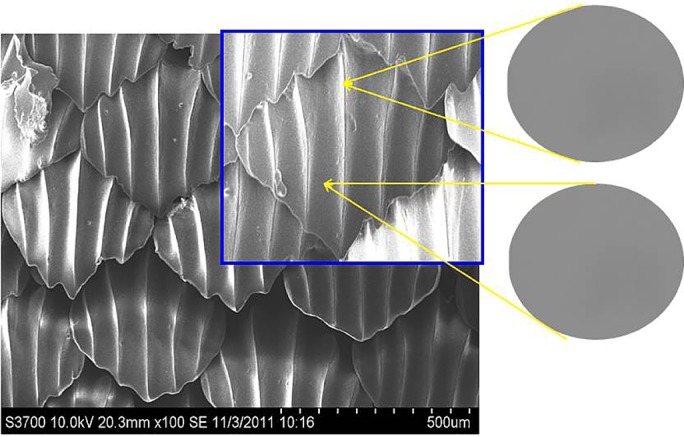
**SEM images of the surface of biomimetic shark skin prepared.** The biomimetic shark skin possessed almost the same surface microstructure as that of the dermal denticles on the real shark skin, which is shown in Fig.1. The high-magnification SEM images showed that the surface of the biomimetic shark skin were very smooth and did not contain any nanostructured protuberances. Thus, micron-scale surfaces can only be realised using bio-replicated forming techniques.

The SEM images of the surfaces of the real shark skin and those of the PU sheet samples prepared by the microreplication technique are shown in Figs 1 and 2. Compared with the real shark skin in Fig. 1, the shark skin-like polyurethane (S-PU) sample exhibited almost the same surface microstructure as that of the dermal denticles on the real shark skin. This observation indicates that the surface morphology of the dermal denticles on shark skin can be simulated by means of replication techniques. In this replication process, the properties of polydimethylsiloxane (PDMS) and PU as a moulding material play an important role. Due to the excellent flow properties of the PDMS and PU precursors, the interstices of the mould can be adequately filled. The subsequent cross-linking process transformed the fluid filling precursor into a solid sheet, while maintaining a conformal contact with the mould cavity and faithfully replicating the fine structure of the mould. The relatively low surface free energy of PDMS and the elasticity of the cured PDMS and PU allowed the prepared sheet to be easily demoulded.

According to previous research (Su et al., 2008; Pu et al., 2016), nanostructured protuberances exist at the riblet valleys of the shark skin surface. The observed riblet peaks were relatively smooth (shown in Fig. 1). However, the surface of the shark skin-like PDMS (S-PDMS) was very smooth and did not contain any nanostructured protuberances (shown in Fig. 2). Thus, micron-scale surfaces can only be realized using bio-replicated forming techniques.

### Surface wettability of biomimetic shark skin

**Fig. 3.**
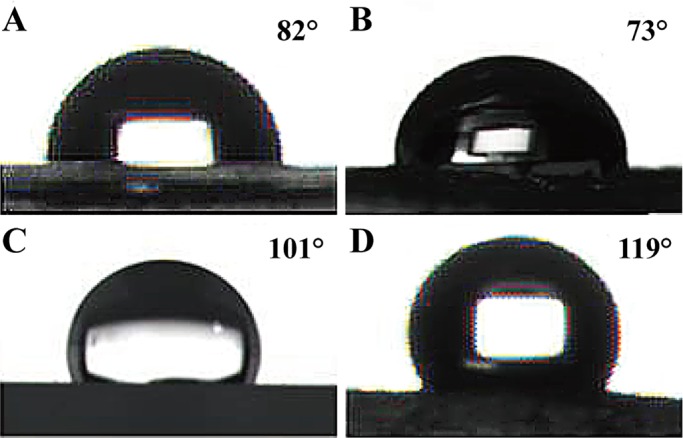
**Droplet contact angles for different surfaces.** (A) A flat sheet was designated as F-PU, (B) a PU sheet with the microstructure of the shark skin surface was designated as S-PU, (C) a flat PDMS was designated as F-PDMS F-PDMS, and (D) a PDMS sheet with the microstructure of the shark skin surface was designated as S-PDMS.

The hydrophobicities of the various microstructured surfaces of the PDMS and PU sheets were evaluated by measuring the respective water contact angles (CAs). The tested samples included the flat PU (F-PU) and flat PDMS (F-PDMS) sheets, which had smooth surfaces, as well as the S-PU and S-PDMS sheets, which exhibited well-defined shark skin morphologies. The results, shown in Fig. 3, revealed that the CAs for the smooth surfaces of F-PU and F-PDMS were 82° and 101°, respectively. The images also show the hydrophilicity of the (–NHCOONH–) bond and the hydrophobicity of PDMS, which is a material with a low surface energy. Additionally, as shown in Fig. 3B, the CA on the surface of the S-PU with microstructures was found to be at 73° relative to the F-PU. Thus, the S-PU was more hydrophilic than the F-PU. However, the results also revealed, as shown in Fig. 3D, that the CA for the microstructured surface of the S-PDMS was 119°, which indicates that the S-PDMS surface was more hydrophobic than the F-PDMS surface.

### Anti-biofouling properties of the biomimetic shark skin surfaces

In this study, the adhesion property of hydrophobic membrane material with biomimetic shark skin surface microstructure (S-PDMS) was investigated by evaluating algal cell and protein adhesion. Algal cell adhesion was observed by SEM, while protein adhesion was observed and analysed indirectly by evaluating the changes of surface CAs.

### Anti-algal adhesion properties

**Fig. 4.**
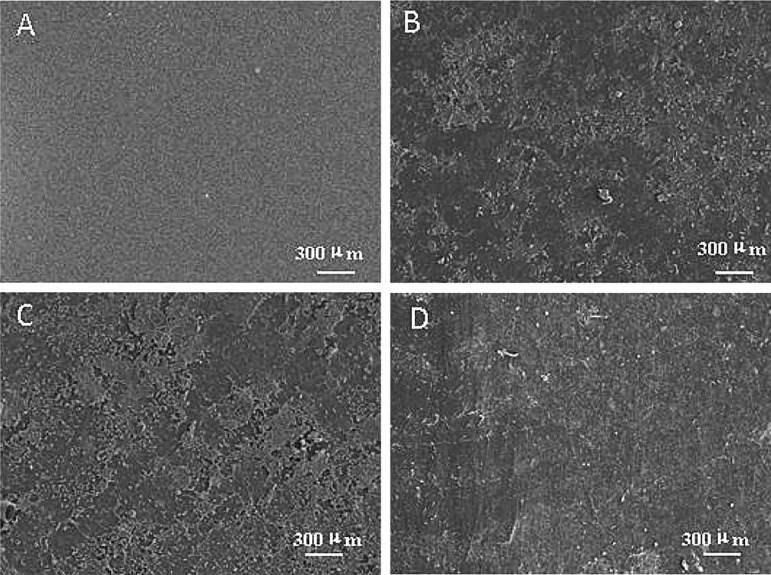
**SEM images of algal cells adhered onto the surfaces of F-PDMS sheet after being soaked in the Pearl River for different times.** (A) 1 day, (B) 21 days, (C) 70 days, (D) after being washed.

**Fig. 5.**
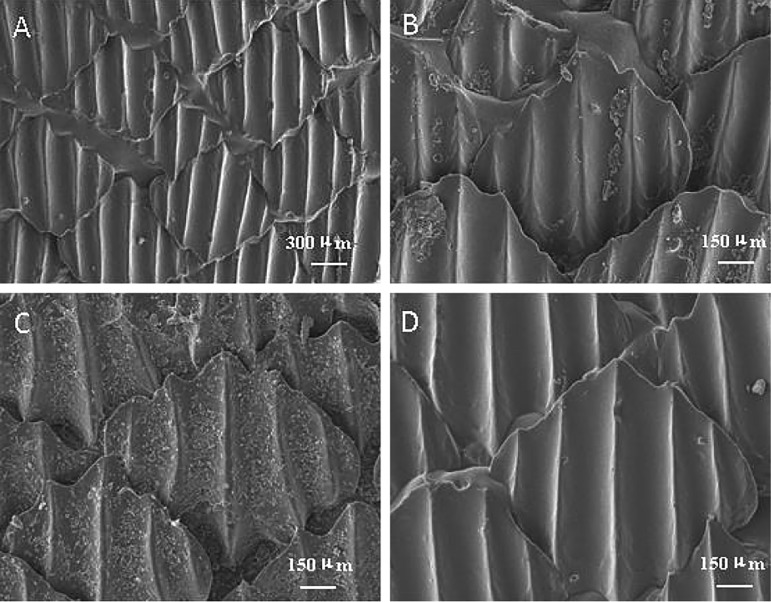
**SEM images of algal cells adhered onto the surfaces of the biomimetic shark skin S-PDMS after being soaked in the Pearl River for different times.** (A) 7 days, (B) 21 days, (C) 70 days, (D) after being washed.

The adhesion of algal cells to surfaces during different periods of time is shown in Figs 4 and 5 for the F-PDMS with smooth surface and S-PDMS with microstructured surface. According to these results, the surfaces of both membrane materials exhibit good anti-biofouling properties after soaking in seawater for 21 days. In fact, there was only a small amount of algae adhered in the local area of surface groove in the bottom for S-PDMS. By contrast, after soaking in seawater for 70 days, it was found that the surfaces of the both membrane materials were covered with plenty of algae because the resistance to wettability was reduced after such a prolonged soaking, and the anti-biofouling property was gradually lost. Nevertheless, after examining and comparing these two figures, it was clear that while the surface of S-PDMS membrane material was covered with algae, the adhesion thickness and quantity of algae adhered to it were smaller than those of F-PDMS. Indeed, after washing with water, there was still a small amount of residual algae on the surface of F-PDMS, whereas the surface of the S-PDMS membrane material was smooth, and algae detached from the surface easily. Besides, the mark left by the algae was invisible. The results of anti-algae performance test on hydrophobic surface-layer F-PDMS and S-PDMS revealed that the anti-biofouling property of the surface of S-PDMS was superior to that of F-PDMS.

### Anti-protein adhesion

**Fig. 6.**
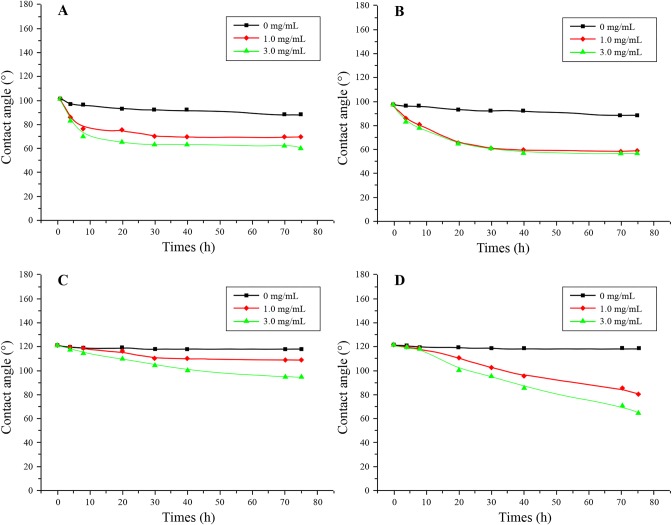
**Changes of the water contact angle of the different surfaces.** Contact angle was calculated over different soaking times and with different concentrations of BSA (A,C) and OVA (B,D) protein solutions for F-PDMS (A,B) and S-PDMS (C,D).

The liquid environment where algae grow contains a large number of organic molecules, such as polysaccharides, proteins and glycoproteins, etc. On the other hand, living organisms, such as bacteria and algae can also secrete biological substances, such as extracellular polymeric substances consisting of polysaccharides, protein polysaccharides and protides on the surface of the material. Thus, in this study, bovine serum albumin (BSA) and ovalbumin (OVA) were used as model protein and glycoprotein, respectively, to study the surface anti-protein adhesion property of S-PDMS. The CA curves of the F-PDMS and S-PDMS surfaces tested after soaking for various time periods, at different concentrations of BSA or OVA, are shown in Fig. 6, including the CAs of various samples, tested in deionized water, which were used as reference. The CA variation diagram after the surface was coated with BSA protein under different conditions is presented in Fig. 6A,C. Where it can be seen (Fig. 6A) that the CA on the surface of F-PDMS gradually declined with time and increasing concentration of BSA protein, and it reached a stable state. However, the CA declining trend was not significant when the concentration of protein was higher. In comparison, on the surface of S-PDMS (Fig. 6C), when the concentration of protein was 1.0 mg/ml, the CA on its surface declined relative to the control samples. However, after soaking for over 72 h, the surface CA was still up to 110°, which indicates good surface hydrophobic stability. Meanwhile, with increasing protein concentration, the surface CA inordinately exhibited different downtrends, and when the concentration of protein was higher, the surface CA declined faster. In addition, the variation trend of the different samples soaked in OVA solution (Fig. 6B,D) for various time periods was approximately similar to that seen with BSA solution (Fig. 6A,C).

### Drag reduction performance of the biomimetic shark skin surfaces

**Fig. 7.**
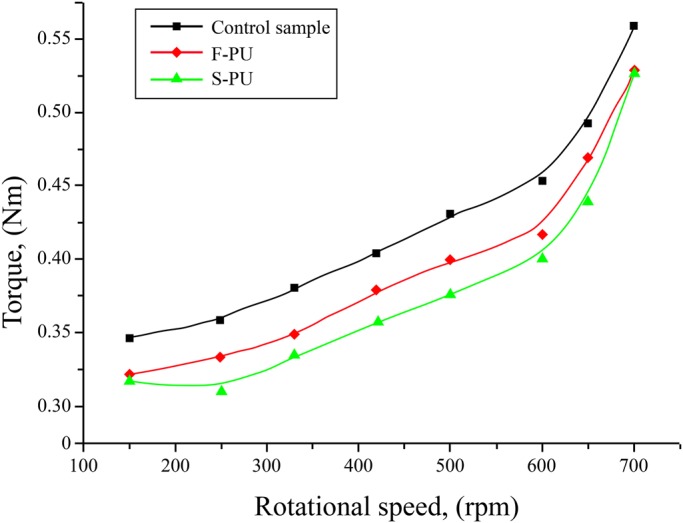
**The torque-rotational speed curves of F-PU and S-PU.** In this experiment, the rotational speed range of rotor is 0∼700 rpm. Glycerin solution was served as fluid medium, its density is 1.255 g/cm^3^, and viscosity is 9.45 P at 20°C.

Torque-rotational speed curve was obtained by testing the drag reduction performances of F-PU membrane material which had smooth surface and S-PU membrane material which had surface micro-structure at the thickness of 2.0 mm, as shown in Fig. 7. The analysis result of drag reduction rate was shown in Table 1. It can be seen from Fig. 7 that the torque of S-PU membrane material that has shark skin surface micro-structure was not only less than the control sample, but also less than the corresponding torque of smooth-faced F-PU membrane material in the testing range. It can be seen from the analysis result of drag reduction rate on the samples that the maximum drag reduction rate of S-PU was 12.5%, which is higher than the corresponding maximum drag reduction rate of smooth-faced F-PU membrane material. Besides, when it reached the rotational speed at 500 rpm, a preferable drag reduction effect could be maintained.

**Table 1.**
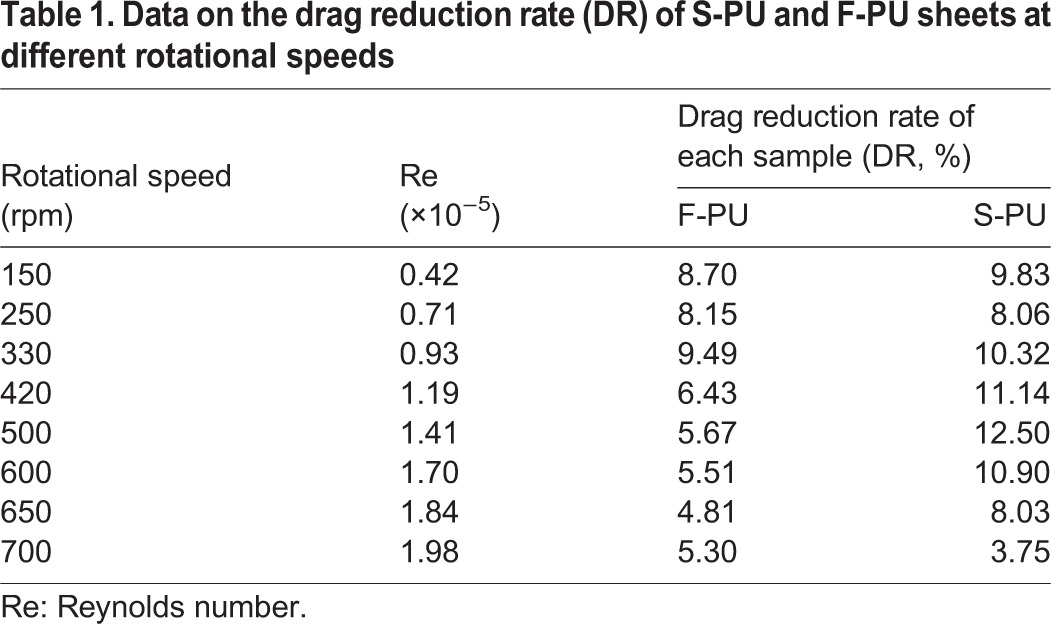
**Data on the drag reduction rate (DR) of S-PU and F-PU sheets at different rotational speeds**

## DISCUSSION

### Impact of biomimetic shark skin surface microstructure on surface wettability

**Fig. 8.**
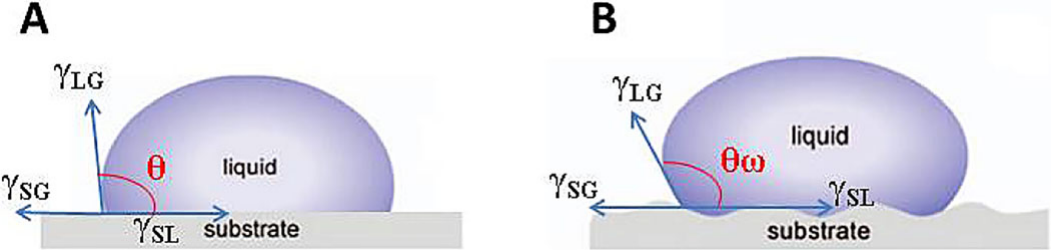
**Surface tension in a droplet on a flat and a rough surface.** Wherein γ_LG_ is the surface tension between the liquid area and gas area; γ_SG_ is the surface tension between the solid area and gas area; γ_SL_ is the surface tension between the solid area and liquid area; θ and θ_w_ are the equilibrium CA of the flat surface (A) and the roughened surface (B), respectively.

Surface wettability has been found to be closely connected to the chemical nature and topology of the surface, and previously have indicated that, comparing with the smooth surface, the microstructured surface of shark skin can improve the hydrophobic feature (Zhao et al., 2012; Liu and Li, 2012). However, based on our studies about surface wettability, comparing with the smooth surface, the PDMS-based microstructured surfaces presents more hydrophobic, and the PU-based microstructured surfaces presents more hydrophilic. The Wenzel model was used to relate the aforementioned phenomenon to the hydrophobicity or hydrophilicity and the surface roughness due to the fabricated microstructure. When water drops were dispensed onto the solid microstructure surface, the liquid completely filled the gaps on the rough surface. This Wenzel contact state (Wenzel, 1936) is shown in Fig. 8, while the Wenzel model is given in Eqn 1 below (Wenzel, 1949):1
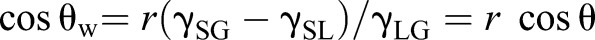
Wherein *r* is defined as a constant roughness factor, which is a dimensionless ratio between the solid surface area and the nominal surface area; cos θ_w_ is the equilibrium CA of the roughened surface. This equation shows the following: (1) when θ<90°, θ_w_ decreases as the surface roughness increases, such that the surface becomes more hydrophilic; (2) when θ>90°, θ_w_ increases with the surface roughness, resulting in a more hydrophobic surface. Accordingly, the S-PDMS with microstructures exhibited a stronger hydrophobicity than the F-PDMS with a smooth surface, where the latter exhibited a CA above 90°. In contrast, the PU surface was itself hydrophilic, with a CA below 90°. Meanwhile, when microstructures were constructed on the smooth surface, the S-PU was more hydrophilic than the F-PU surface.

### Analysis of anti-biofouling properties of the biomimetic shark skin surfaces

Biofouling is the accumulation of living organisms on a surface including bacteria, fungi, protozoa, algae and invertebrates. The growth of marine organisms on ship hulls is a major expense factor in naval industries. PDMS, low surface energy silicone material, was a nontoxic alternative to conventional biocide paints. While PDMS is not inherently antifouling due to the release of hard fouling and soft fouling under suitable hydrodynamic conditions, and bioaccumulation will occur under static and low flow conditions (Holm et al., 2006), in recent years, the approach to reduce organism settlement on PDMS surface utilizes engineered surface topographies, including a unique pattern ‘Sharklet AF™’ similar to shark skin surface (Schumacher et al., 2007). In this paper, a biomimetic PDMS-based surface that is highly similar to a shark skin surface was prepared using PEES method (a bio-replicated forming technique). Anti-biofouling properties of these biomimetic surfaces were firstly studied.

According to the previous results about anti-biofouling properties (Xu et al., 2014), in a biological adhesion environment, the hydrophobic surface of biomimetic shark skin has excellent antibiont adhesion property during a certain time period, which is related to the special chemical compositions and microstructures of the surface. As the surface of S-PDMS has a special microstructure, the surface free energy is low, and the air layer is hidden in the surface structure; factors which reduced the interaction site of the surface and adhesion organism. Besides, if not firmly adhered, the adhesion organism can be easily washed out. Thus, it provides an excellent antibiont adhesion effect. However, when the surface of S-PDMS is soaked in a liquid environment with bioactive substances, the action sites between the surface and external biological matrix are increased due to the loss of surface air layer. Consequently, the effect of antibiont adhesion is lost. In addition, it can be seen that the air layer fixed on the surface of S-PDMS plays a key role in the surface where there is antibiont adhesion property.

The surface of S-PDMS is a liquid environment containing a bioactive substance. The reason why the surface air layer gradually disappears with the extension of time may be related to the action of biomolecules like proteins. This result can be explained taking into account the protein characteristics, the surface chemical compositions of different materials and the analysis of microstructure. Thus, on the smooth surface of F-PDMS, where there is low surface energy, proteins like BSA and OVA can be easily delivered onto the surface of the material in the solution. The chemical property on the surface of the F-PDMS material changes due to the interaction on the surface, which results in adsorption, rearrangement and permanent adhesion and causes changes in contact angle. In comparison, on the surface of the S-PDMS material the thin air layer adsorbed by surface the microstructure forms a physical-absorption protein barrier which decreases interaction sites with protein molecule. As a result, the protein cannot be successfully delivered to the surface, thus showing an anti-protein adhesion effect. However, with the increase of protein concentration and the extension of the contact time with the interface, the surface tension of the solution can be changed with the increase of protein concentration. On the other hand, protein may gradually become adhered to the previous few contact sites, which progressively increases the contact area between the protein solution and the material surface. Thus, the air layer physical-absorption barrier adsorbed onto the material surface microstructure is gradually destroyed until the air layer disappears completely. Finally, the surface CA declines due to the large adhesion of protein, and the hydrophobic characteristic of the material itself disappears. The result above fully shows that the thin air layer of the microstructure on the surface of S-PDMS, by acting as a physical barrier, can play an important role in antibiont adhesion.

### Drag reduction of biomimetic riblet-structured surfaces

It can be seen from the analysis on the drag reduction performance of smooth-faced F-PU membrane material and the S-PU membrane material which has riblet-structured surfaces that these two membrane materials had drag reduction effect in the investigated range of Reynolds number (0.42×10^5^∼1.98×10^5^), and S-PU membrane material showed a more significant drag reduction effect. Based on the experimental results, biomimetic riblet-structured surfaces is conducive to improving the drag reduction performance of homogeneous and flexible PU sheet. The structure of micro-riblets formed by PEES method leads to the higher value of drag reduction rate which is proven by the drag reduction experiments. The reason is because that the surface of PU sheet can replicate the riblet structure of real shark skin accurately, which gives full play to the inhibition on the micro-structure surface of shark skin and delay turbulence to happen. Besides, the non-smooth surface of flexible S-PU sheet constitutes an organic integrity with the matrix flexibility and has a synergetic drag reduction effect on the frictional resistance of fluid.

Studies on the drag-reduction behavior of different biomimetic riblet-structured surfaces have been reviewed [Bhushan, 2009; Dean and Bhushan, 2012; Lang et al., 2014]. Results show that compared with flat surfaces as control specimens, the investigated biomimetic surfaces with riblet structures, with cross-section shapes that include sawtooth, scalloped, blade, trapezoidal, semi-circular, and rectangular shapes, can reduce the drag in fluid flow, which usually ranges from 5% to 10% (Bechert et al., 1997, 2000). The drag-reduction performance of these biomimetic non-smooth surfaces depends on numerous factors, such as riblet geometries, characteristics of the used matrix, test methods for evaluating the drag-reduction effect, and specific test conditions. Accordingly, it is difficult to evaluate data on the drag-reduction effect of different biomimetic non-smooth surfaces through simple comparison.

## CONCLUSIONS

In this study, the microstructures of the shark skin surface were replicated on the surface of PDMS and PU sheets by the PEES method. SEM observations revealed the well-defined shark skin surface morphologies or micro-sized shark skin pattern structures of the biomimetic shark skins. The Wenzel model of hydrophobic theory and the water CA values of the different microstructured surfaces of the PDMS and PU sheets showed that the microstructures affected the surface wettability. Furthermore, the anti-algae performance test showed that the hydrophobicity of the material and surface microstructure can together block the adhesion of marine organisms. Additionally, the anti-biofouling property of S-PDMS with micro-structure surface was superior to F-PDMS with smooth surface. The thin air layer of the microstructures on the surface of S-PDMS can play an important role in antibiont adhesion by acting as a physical barrier. Based on the drag-reduction experimental results, the maximum of drag reduction rate of the biomimetic shark skin prepared by PEES method is 12.5%, which is higher than the corresponding maximum drag reduction rate of membrane material with smooth surface. This suggested that the micro-structured surface of the biomimetic shark skin prepared by PEES method played a significant role in reducing drag.

Although we have fabricated the same surface microstructure as that of the dermal denticles on the real shark skin, we were not able to capture the fine nanostructured protuberances present on the surface of the shark skin, and only the microstructured surfaces could be produced. We anticipate that a notable progress in anti-biofouling and drag reduction will be accomplished using biomimetic applications of superhydrophobic surfaces at the riblet valleys, the riblet peaks, or alternatively across the entire surface. Additionally, the mucous substance naturally present on the skin of sharks has been proven to cause a reduction in drag or bio-adhesion as they move through the water. Thus including such substances with similar properties is a desirable feature. We also expect that both, in combination and separate, the mucus and surface-roughness characteristics of the shark skin will improve efficacy compared with current fabricated surfaces.

## MATERIALS AND METHODS

### Materials

Fresh shark skin from one of the fastest swimming sharks, the great white shark (*Carcharodon carcharias*), was purchased from a fisherman. The room temperature vulcanizable liquid silicone rubber used consisted of two components, a low-velocity vinyl-polydimethylsiloxane (PDMS) precursor and a curing agent and was obtained from Zhejiang Runhe Silicone New Material Co., Ltd (Zhejiang province, China). The PDMS contained 25% of a fumed silica (300 m^2^/g) filler, which was treated with Si(Me)_2_-O-oligomers. A two-component room temperature vulcanizable liquid polyurethane (PU), comprising a precursor and curing agent, was used to fabricate a biomimetic shark skin surface. The precursor with a 4.8% isocyanate (NCO) content was prepared by reacting diisocyanate with polytetramethylene ether glycol. The formaldehyde, alcohol, and acetone reagents were all analytical grade.

### Fabrication of biomimetic surfaces based on shark skin

**Fig. 9.**
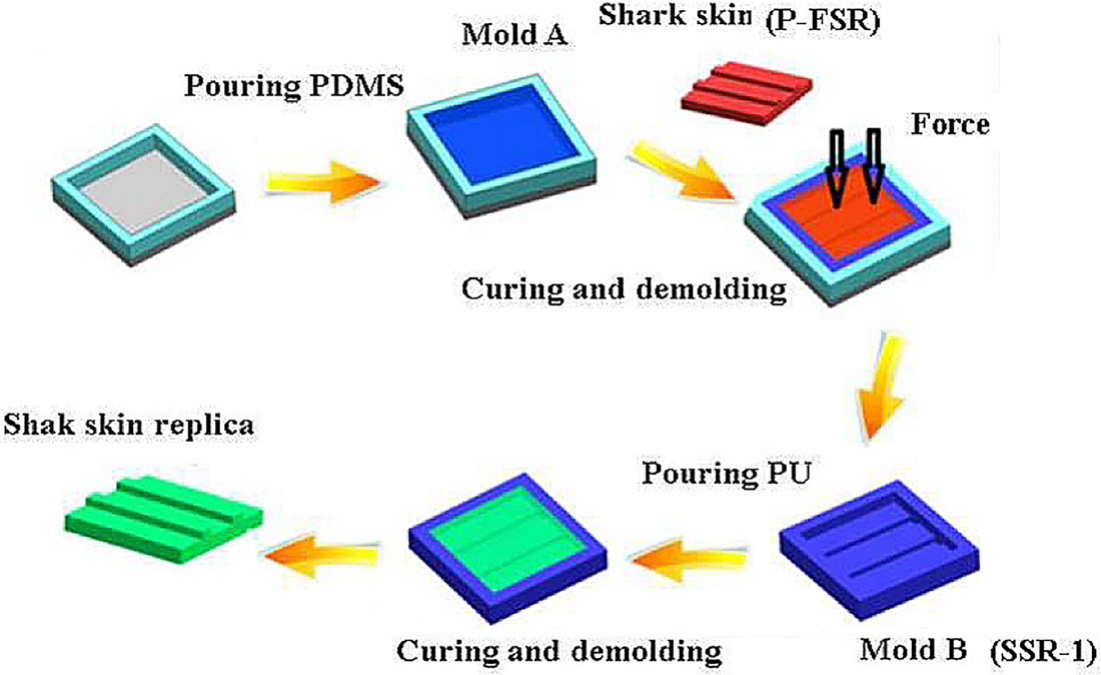
**Schematic diagram of the microreplication process of the shark skin surface.** FSR means fresh shark skin. P-FSR means the FSR that was processed with the cured PDMS elastic matrix. SSR-1 means PDMS-based shark skin replica. This replica was served as a negative mold.

The microstructure of the shark skin surface can be replicated onto the surface of PDMS and PU sheets by the PDMS embedded elastomeric stamping (PEES) method. Specifically, the process involves, as shown in Fig. 9, the following steps: (1) A sheet of fresh shark skin was fixed onto a plate, and oven-dried at 40-60°C for 1 to 2 h. These skins were designated as fresh shark skin (FSR). To prevent the skins from shrinking and warping, a mixture of the PDMS precursor and curing agent (10:1 by weight), was layered at a thickness of approximately 1 mm and flattened at the back of the FSR, which was then cured at room temperature for 2-3 h. The FSR that was processed with the cured PDMS elastic matrix was designated as P-FSR. (2) A mixture of the PDMS pre-polymer and the curing agent (10:1 by weight) was first poured into a plate and evacuated in a vacuum oven for 2-10 min. Subsequently, the shark skin template (P-FSR) with its scale side down was carefully embedded onto the surface of an uncured PDMS sheet. Then, isostatic pressure was applied to the P-FSR to transfer the surface microstructures of the shark skin to the PDMS surface in contact with the shark skin. After curing at room temperature for 30 min, the cured PDMS sheet for microreplication was separated from the P-FSR as the desired shark skin replica (SSR-1). The SSR-1, with a counter-shape of the shark skin pattern, served as a negative mould. (3) The PU pre-polymer and the curing agent were mixed and degassed in a desiccator, which was then poured onto the surface of the P-FSR. After curing and demoulding, the PU sheet with the microstructure of the shark skin surface (S-PU) was then completely fabricated. A PDMS sheet with a shark skin surface microstructure (S-PDMS) was fabricated using the same microreplication process as in the preceding step. A mixture of the PU pre-polymer and the curing agent was poured into a plate and cured to obtain a flat PU sheet, which was designated as F-PU. A flat PDMS sheet was also prepared using the same method and designated as F-PDMS.

### Measurement and characterisation

#### Surface characterization

##### Examination of surface morphology

The surface morphology of shark skin and those of the prepared PDMS and PU sheets were observed by SEM using a S-3700N scanning electron microscope (Hitachi, Japan) and a field emission scanning electron microscope (LEO 1530 VP, Elektronenmikroskopie GmbH Oberkochen, Germany). The sample surfaces to be observed were gold-coated using a sputter coater beforehand.

##### Measurement of contact angle

The contact angle (CA) was measured using a DSA 100 contact angle goniometer (Krüss GmbH, Germany) at ambient temperature. Each sample was measured three times at three random locations, and the average of the measured values is reported.

#### Antifouling performance test

##### Protein adhesion experiment

Bovine serum albumin (BSA) and ovalbumin (OVA) were prepared at two different concentrations, at 25°C. The samples to be tested were soaked in the BSA/OVA protein solution. After the soaking was complete, the surface was slightly rinsed with distilled water and the non-adhered protein was washed off. Finally, the surface of the samples adhered to proteins was dried at room temperature to perform the contact angle test.

##### Anti-algae performance test

The antifouling property on the biomimetic shark skin surface was also characterized through an anti-algae performance test. The size of the membrane material tested was 6×20 cm. The membrane material was pasted flatly on a hard board. The board was then fixed in a plastic basket vertically, which was immersed into the ocean and fixed at a depth of 0.5-1 m. Samples were taken out every 7 days, and the surface was washed. At the same time, the growth of algae on the surface of the membrane material was observed by SEM. The testing place was in the Zhujiang water area, Haizhu District, Guangzhou, China, and the testing time was from November 15th, 2013 to April 10th, 2014.

### Fluid-drag measuring experiment

**Fig. 10.**
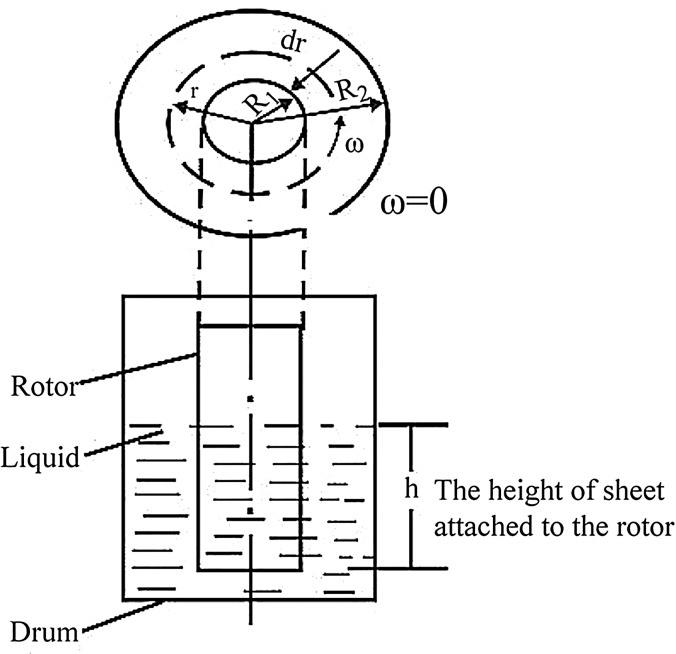
**Schematic diagram of the drag measuring experiment.** In this diagram, the rotor radius *R_1_* is 0.06 m, and the drum *R_2_* is 0.105 m, the height of the sheet attached to the rotor is 0.06 m. The rotational speed range of rotor is 0∼1000 rpm.

In order to evaluate the replication quality of biomimetic shark skin accurately, it was necessary to do a fluid-drag measuring experiment. JZY-009 resistance tester was applied to have drag-reduction performance test on the samples. Resistance tester was designed and produced according to the basic principle of rotational viscometer testing method. The schematic diagram of drag measuring experiment was demonstrated in Fig. 10.

The samples to test, F-PU and S-PU, were made into an appropriate size of 20 cm×5 cm and applied to the surface of rotor. The rotor is adjusted to the speed set, measuring and recording the torque M of rotor at this speed set on the digital indicator. Reynolds number and drag reduction rate were calculated at this rotational speed. The rotational speed range of rotor is 0∼800 rpm in this experiment, and the temperature is 20°C to test fluid medium. The density of glycerin solution is 1.255 g/cm^3^, and viscosity is 9.45 P.

The Reynolds number (Re) associated with the rotating drag-measure instrument is defined as:2
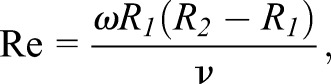
where *R_1_* is the rotor radius (m) and *R_2_* is the water bath radius (m), *ω* is angular velocity (rad/s), *ν* is the kinematic viscosity of water (m^2^/s).

The drag reduction rate is defined as:3
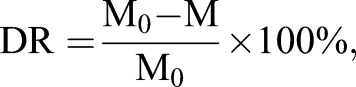
where M_0_ is the torque of the rigid disc (Nm) and M is the torque of the disc affixed sample at the same rotational speed (Nm).
